# Synchrotron X-ray microtomography and multifractal analysis for the characterization of pore structure and distribution in softwood pellet biochar

**DOI:** 10.1007/s42773-021-00104-3

**Published:** 2021-06-23

**Authors:** Franziska Srocke, Liwen Han, Pierre Dutilleul, Xianghui Xiao, Donald L. Smith, Ondřej Mašek

**Affiliations:** 1 Department of Plant Science, Macdonald Campus, McGill University, 21111 Lakeshore Road, Ste-Anne-de-Bellevue, Quebec, H9X 3V9 Canada; 2 UK Biochar Research Centre, School of Geosciences, University of Edinburgh, King’s Buildings, Alexander Crum Brown Road, Edinburgh, EH9 3FF UK; 3 Advanced Photon Source, Argonne National Laboratory, Lemont, IL 60439 USA; 4 Present Address: National Synchrotron Light Source II, Upton, NY 11973 USA

**Keywords:** Biochar, Pore structure, X‐ray microtomography, Homogeneity, Isotropy, Multifractal analysis

## Abstract

Biochar pores in the micrometer range (1–100 µm) derive from cellular structures of the plant biomass subjected to pyrolysis or can be the result of mechanical processing, such as pelleting. In this study, synchrotron X-ray microtomography was used to investigate the internal pore structure of softwood pellet biochar produced by slow pyrolysis at 550 and 700 °C. The microtomographic data sets consisted of 2025 images of 2560 × 2560 voxels with a voxel side length of 0.87 µm. The three-dimensional reconstructions revealed that pelleting and pyrolysis significantly altered the pore structures of the wood feedstock, creating a network of connected pores between fragments that resembled the wood morphology. While higher pyrolysis temperature increased the specific surface area (as determined by BET nitrogen adsorption), it did not affect the total observed porosity. Multifractal analysis was applied to assess the characteristics of the frequency distribution of pores along each of the three dimensions of reconstructed images of five softwood pellet biochar samples. The resulting singularity and Rényi spectra (generalized dimensions) indicated that the distribution of porosity had monofractal scaling behavior, was homogeneous within the analyzed volumes and consistent between replicate samples. Moreover, the pore distributions were isotropic (direction-independent), which is in strong contrast with the anisotropic pore structure of wood. As pores at the scale analyzed in this study are relevant, for example, for the supply of plant accessible water and habitable space for microorganisms, our findings combined with the ability to reproduce biochar with such pore distribution offer substantial advantages in various biochar applications.

**Supplementary Information:**

The online version contains supplementary material available at 10.1007/s42773-021-00104-3.

## Introduction

Biochar is the solid porous, carbon-rich product formed during pyrolysis of biomass at temperatures above 350 °C under oxygen-limited conditions. Common lignocellulosic biomass feedstocks for biochar production include forestry and agricultural wastes, including wood chips, tree bark, straw, nut shells and rice husks (Sohi et al. 2009). Incorporation of biochar into soil can improve soil properties and functions, such as nutrient and water retention, and simultaneously achieve atmospheric carbon sequestration. Moreover, the use of biochar as an adsorbent for contaminant removal from wastewater or as porous medium in biofilters has been receiving significant attention recently (Baltrėnaitė et al. 2017; Huang et al. 2019; Shaheen et al. 2019). The specific application of biochar depends on its physical and chemical properties, which, in turn, are determined by the feedstock properties and conversion process parameters (Weber and Quicker 2018; Zhao et al. 2013). Thus, a detailed understanding of the transformation of biomass during pyrolysis and the impact of pre- or post-pyrolysis treatments is required for the tailored production and optimization of biochar properties. This includes effects on biochar surface area, porosity and internal structure, which can influence physical processes in the environment or in technical applications, such as the transport and storage of water in soil or gas diffusion in biofilters.

The pore space in biochar is complex, with pore sizes spanning at least five orders of magnitude, from sub-nanometer micropores to macropores of tens of micrometers (Brewer et al. 2014; Gray et al. 2014). During pyrolysis, volatiles are released from biomass and micro- (< 2 nm) and mesopores (2–50 nm) are formed. This so-called pyrogenic porosity contributes marginally to the total porosity but adds considerably to the surface area of biochars. With higher pyrolysis temperatures, the surface area increases as more micropores are created, until a maximum surface area is reached (Brown et al. 2006; Chia et al. 2015).

In plant-based biochars, large pores in the range of 1–100 µm originate from the cellular plant structures and contribute most to the total pore volume (Gray et al. 2014). Due to the regular morphology and size of plant cells, macroporosity in wood chars is comprised of discrete groups of pore sizes rather than a continuum (Wildman and Derbyshire 1991). Wood tissue consists mostly of dead and hollow cells that are arranged in a regular pattern. In softwood, longitudinally oriented tracheids account for 90–95% of cells, are generally about 35–50 µm wide and 3–5 mm long and connected through small openings known as pits (Parham and Gray 1984). Radially oriented parenchyma cells are called rays, which contain horizontal resin canals in some softwood species (Parham and Gray 1984). The cell structures of the original wood are still recognizable in pyrolyzed wood, but thermal decomposition causes shrinkage and deformation of cell walls, with the result that pores appear irregularly shaped with rougher surfaces as pyrolysis temperature and heating rates are increased (Bird et al. 2008; Hyväluoma et al. 2018a; Wildman and Derbyshire 1991; Zeng et al. 2015).

Biochar macroporosity has the potential to influence soil hydrologic properties by altering volume, size, distribution and connectivity of pore spaces in soil (Masiello et al. 2015). Soil pores with diameters of 50–500 µm are responsible for transport of water and air, while pores of 0.5–50 µm store water that is available to plant roots (Greenland 1977; Pagliai and Kutilek 2008). Several studies have reported that biochar improved field capacity and plant-available water content, especially in sandy soils (Abel et al. 2013; Aller et al. 2017; Edeh et al. 2020; Hansen et al. 2016; Rasa et al. 2018). Moreover, micrometer-range biochar pores can be inhabited by microorganisms, e.g., soil bacteria and mycorrhizal fungi, provided that the pores are not blocked by adsorbed organic compounds or condensed volatiles (Pietikäinen et al. 2000; Quilliam et al. 2013; Thies et al. 2015). Although microbial cell sizes can range between 0.2 and 750 µm in diameter across taxa, Portillo et al. (2013) found that more than 60% of archaeal and bacterial cells extracted from soil were smaller than 1.2 µm in diameter. Thus, plant-derived micrometer-range pores in biochar should be large enough to offer habitable space for most soil microbes.

Surface area and volume of pores < 50 nm are typically analyzed by carbon dioxide or nitrogen gas physisorption with Brunauer–Emmett–Teller (BET) analysis (Rouquerol et al. 1994; Sun et al. 2012). These parameters are valuable for the characterization of physical properties and environmental behavior of biochar, for example, as adsorbent of various contaminants. Although the N_2_ physisorption measurement with BET is a well-established technique, it can lead to unreliable results when applied to biochar due to the physical and chemical complexity of the material, and modifications to the method have been proposed recently (Maziarka et al. 2021; Sigmund et al. 2017). For the characterization of a wider range of pore sizes, mercury intrusion porosimetry is widely employed for the measurement of porosity, specific surface area, and the distribution of pore opening sizes ranging between 3 nm and 400 µm (Rouquerol et al. 2012). In addition, fluid displacement measurement (pycnometry) of total porosity and density has been shown to be a suitable technique for biochars, which yields pore volumes similar to the values obtained by mercury porosimetry (Brewer et al. 2014). Besides the estimation of porosity and pore sizes, computed X-ray microtomography allows three-dimensional (3D), non-destructive and quantitative analysis of pore geometry, distribution and connectivity within the carbon matrix. It has been successfully applied to biochars and wood chars with a resolution between 0.65 and 5.67 µm voxel size, depending on the equipment used, which allows the investigation of pores larger than those captured by gas adsorption techniques, and which are relevant for soil hydraulic properties, microbial colonization and wood char gasification (Berhanu et al. 2018; Conte and Nestle 2015; Hyväluoma et al. 2018a, b; Jeffery et al. 2015; Jones et al. 2015; Pattanotai et al. 2014, 2015; Schnee et al. 2016; Watanabe 2018). The reconstructed 3D images of biochars in these studies illustrated well-preserved cellular structures of the original biomass. For instance, Berhanu et al. (2018) observed that the pore morphology of pyrolyzed softwood resembled that of the original wood, and mainly consisted of interconnected longitudinal pores with mean diameter of about 20 µm. The authors also found that pyrolysis temperature (400 vs. 700 °C) did not affect the total observed macroporosity. Hyväluoma et al. (2018a) reported that the micrometer-range pore structure in willow biochar was determined by the vascular tissue structure of the wood and, therefore, had a high degree of anisotropy. Pyrolysis (at 308, 384, 489 °C) caused shrinkage and changes in pore shapes and pore size distribution but did not alter the total porosity estimated from microtomographic data sets.

In contrast to the wood biochars previously characterized by X-ray microtomography, our work focused on biochar produced from pelletized wood. In 2019, a total of 298,000 tonnes of wood pellets and briquettes were produced in the UK, and 8.9 million tonnes of wood pellets were imported into the UK, mainly from the USA (62%) and Canada (18%) (Forestry Commission 2020). The majority of pellets is used for heating or power generation. Milling and pelleting of biomass creates particles of uniform shape, size, and density from materials that are inherently heterogeneous and of low bulk and energy density. Consequently, this physical pre-treatment reduces the biomass variability, for example, in particle size, morphology and microstructure, which may impact the heat and mass transfer during thermochemical conversion processes, such as pyrolysis (Ciesielski et al. 2018). Additional benefits of pelletizing are that the transport and storage of densified biomass is more efficient, and that pellets are more flowable, allowing them to be continuously fed into the pyrolysis unit (Mašek et al. 2016; Tumuluru 2018). The mechanical pre-treatment for size reduction and densification significantly alters the internal structure and pore geometry of the biomass. Thus, the aim of this study was to investigate the porous structure in biochars produced from softwood pellets by slow pyrolysis at 550 and 700 °C, using X-ray synchrotron microtomography. We tested the hypothesis that pyrolysis temperature does not affect the observed porosity, which in this study includes pores larger than two voxels (1.74 µm), and that pellet biochars have homogeneous and isotropic pore distributions, which would mean that there are no multiple scale dependencies and no preferential direction of variability. We applied the 3D approach for multifractal analysis to quantitatively characterize the pore distribution based on 3D image data sets (Han et al. 2020).

Multifractal analysis has been used to describe scale-dependent self-similar patterns in complex heterogeneous systems, for instance, in porous structures, such as soil and rocks, based on X-ray tomography data sets (Giri et al. 2015; Lafond et al. 2012; Martínez et al. 2010; Wang et al. 2018). Multifractal analysis yields information about the heterogeneity in the variability of the distribution of the studied variable, e.g., the spatial distribution of pores in a solid matrix. A multifractal pore distribution will have several local scaling properties (fractal dimensions), whereas monofractals can be described with a single fractal dimension.

The objectives of this work were to (1) reconstruct the 3D structure of pores in softwood pellet biochars from microtomographic data sets; (2) quantify the observed total porosity, pore volume distribution and pore connectivity; (3) characterize the distribution of pores using multifractal analysis; (4) assess the effect of pyrolysis temperature (550 °C vs. 700 °C) on the observed porosity and pore distribution; and (5) test the hypotheses of homogeneity and isotropy for the distribution of pores.

## Materials and methods

### Biochar production

Biochars were produced from cylindrical softwood pellets 6 mm in diameter (Puffin Pellets, Banff, Scotland) by slow pyrolysis at highest treatment temperatures (HTT) of 550 °C (SWP550) or 700 °C (SWP700), using the pilot-scale rotary kiln pyrolysis unit at the UK Biochar Research Centre (UKBRC) at the University of Edinburgh. Both biochars are part of a set of 12 standard biochars produced with high reproducibility and consistency (Mašek et al. 2018). For the production of SWP550, the temperature at the reactor wall was 550 °C and the mean temperature in the char bed at steady state was 545 °C; the estimated heating rate was 78 °C min^−1^, and the estimated residence time at HTT was 3.9 min. For SWP700, the temperature measured at the reactor wall was 700 °C and in the char bed at steady state 681 °C, the estimated heating rate was 87 °C min^−1^, and the estimated residence time at HTT was 5 min. The biochars retained the characteristic shape of the feedstock pellets. The physicochemical properties of the biochars are summarized in Table 1. Biochar pellets were carefully crushed and particles of approximately 1–2 mm size were randomly selected for scanning electron microscopy and X-ray microtomography.

**Table 1:** Physicochemical properties of softwood pellet feedstock and biochars data from (Mašek et al. 2018); mean values and standard deviation

Parameter	Unit	Softwood pellets	SWP550	SWP700
Moisture	wt%	6.71 ± 0.03	1.52 ± 0.16	1.0 ± 0.24
Volatile matter	wt% d.b	77.92 ± 0.44	14.65 ± 0.98	7.35 ± 1.02
Fixed carbon	wt% d.b	14.22 ± 0.38	84.28 ± 0.93	90.99 ± 0.82
Ash content	wt% d.b	1.07 ± 0.12	1.07 ± 0.41	1.66 ± 0.38
C (elemental)	wt% d.b	49.95	85.52 ± 1.22	90.21 ± 0.39
H (elemental)	wt% d.b	6.65	2.77 ± 0.09	1.83 ± 0.15
N (elemental)	wt% d.b	< 0.1	< 0.1	< 0.1
O (by difference)	wt% d.b	42.23	10.36 ± 1.19	6.02 ± 0.74
H/C molar ratio	–	–	0.39 ± 0.01	0.24 ± 0.02
O/C molar ratio	–	–	0.09 ± 0.01	0.05 ± 0.01
pH	–	–	7.91 ± 0.3	8.44 ± 0.69
Total surface area (N_2_ sorption and BET)	m^2^ g^−1^	–	26.4	162.3

*d.b* dry basis

### Scanning electron microscopy

The surface morphology of biochar was qualitatively examined using a Carl Zeiss SIGMA HD VP Field Emission Scanning Electron Microscopy system at the School of Geoscience, University of Edinburgh. Particles of 1–2 mm size were mounted via adhesive carbon discs onto sample stubs. Coating of the samples was not required as the raw biochar was sufficiently conductive. In addition, whole biochar pellets were embedded in resin, cut transversely or longitudinally, and subsequently coated with carbon for investigation of the internal structure of the pellet. Scanning electron microscopy (SEM) was conducted under vacuum using 20 keV acceleration voltage, with a working distance between 7.0 and 8.2 mm, and an Everhart–Thornley secondary electron detector.

### Synchrotron X-ray microtomography

Synchrotron X-ray microtomography scanning of two SWP550 and three SWP700 replicate particles was conducted at the beamline 2-BM of the Advanced Photon Source at the Argonne National Laboratory, IL, USA. The biochar samples, weighing between 2.3 and 4.8 mg, were placed in a cylindrical ceramic sample holder and fixed on the rotational sample stage at 10 cm from the detector. Attenuated X-rays were collected for 10 s using a pink beam at 27 keV and 5 ms exposure time, while the sample stage rotated from 0 to 180°. For each scan, 900 2D projection images were recorded by a PCO Edge camera (PCO, Kelheim, Germany) with 7.5 × lens magnification and 2560 × 2160 pixels sensor size. The resulting pixel resolution was 0.87 μm.

The 2D projection images were used to reconstruct 3D stacks of 2025 image slices of 2560 × 2560 voxels, using the Gridrec algorithm (Dowd et al. 1999) implemented in the Python-based TomoPy framework (Gürsoy et al. 2014). Prior to reconstruction, ring artifacts were corrected using the combined wavelet-Fourier filter with parameters *L* = 8, *σ* = 4 and wavelet type ‘sym16’ (Münch et al. 2009). Since the carbon matrix of biochar has a weak X-ray absorption contrast, the Paganin algorithm for single-distance phase retrieval (regularization parameter *α* = 0.002) was used to obtain images with enhanced edges between the carbon phase and air-filled pores (Paganin et al. 2002).

### Digital image processing

The image stacks in TIF format were imported to MATLAB R2019a (MathWorks, Natick, MA, USA). The image data (32-bit floating-point, single precision in MATLAB) for each voxel was multiplied by 100,000 and converted into 16-bit signed integers, so that grayscale images could be properly displayed in MATLAB. The histograms of numbers appeared symmetrical (bell-shaped) and centered at 0. Image segmentation was performed by applying a grayscale threshold to assign each voxel in the volume to either pore (void) or biochar solid in the matrix (3D array). After several trials, we found that the selection of voxels with a value greater than 8 (i.e., the use of 8 as threshold) provided a good binarization of pore vs. biochar. Subsequently, only the largest group of inter-connected voxels with the value of one was kept and assumed to represent the entire biochar matrix in the volume. Thereby, unwanted isolated groups of biochar voxels were removed. Regions of interest (ROI) of 1024^3^ voxels were cropped from the center of the binarized volumes, corresponding to sample volumes of about 0.707 mm^3^.

### Analysis of biochar porosity from 3D images

Given the voxel resolution of 0.87 μm, the observed porosity in the context of this study consisted of pores greater than a 1.74^3^ μm^3^ cube. The volume of each observed pore was calculated as the total number of voxels for each pore, and the distributions of estimated pore volumes were presented in histograms (Supplementary material, Fig. S1). Total porosity was defined as the number of voxels of pores with a minimum volume of four voxels, divided by the total number of voxels in the ROI. The volume fraction of the pore with the greatest volume to the total pore volume of the ROI was used as an indicator for pore connectivity (Bird et al. 2008). The surface area of the pore with the greatest volume was measured from its 3D perimeter using the MATLAB function *bwperim*. The surface area-to-volume ratio was calculated by dividing the surface area (expressed in number of voxels) by the volume of the pore (expressed in number of voxels).

### Multifractal analysis

The aim of a multifractal analysis is to find a spectrum of scaling exponents or fractal dimensions that describe how subsets of some measure (in probabilistic sense) with similar local densities vary with scale. The fractal properties of these subsets are described by singularity strengths (Hölder exponents) and generalized fractal dimensions (Rényi dimensions). Their values, once plotted in two types of multifractal spectra, provide information about the variability of the studied variable and the heterogeneity of its distribution, via the spectrum shape.

We performed multifractal analysis on pore distributions using a MATLAB-based application (Han et al. 2020). Equations for the calculation of multifractal parameters are given in ESM Appendix A. The number of segmented pore voxels for each image slice in the ROI was counted and represented in a frequency distribution graph as a function of depth (one of the three perpendicular axes of the cube). The ROI for each of the five samples was analyzed along the three axes of the cube to test whether wood pellet biochar has an isotropic (direction-independent) pore structure, as pore structure is known to be anisotropic (direction-dependent) for non-pelleted wood biochar (Hyväluoma et al. 2018b). This resulted in a total of 15 multifractal analyses. Pore frequency plots were successively partitioned by dyadic downscaling into subintervals [*I*_*i*_(*ε*) (*i* = 1, 2, 3 … 2^*k*^) of length *ε* = 2^−*k*^ *L,* with *k* = 1, …, 10 and *L* = 1024 is the total depth]. Each subinterval covered a contiguous portion of image slices of the ROI, and the measure or mass distribution [*µ*_*i*_(*ε*)] represented the contribution of this portion to the total porosity. Multifractal parameters of singularity strength [*α*(*q*)] and the associated Hausdorff dimension [*f*(*α*_*q*_)] and of generalized dimensions (*D*_*q*_) were estimated for moment orders *q* between − 9 and + 9 in increments of 1. Estimates were kept only if the coefficient of determination *R*^2^ in the fitting of a straight line in the relevant log–log plot was greater than or equal to 0.9. Positive values of *q* > 1 explore subsets with higher concentrations of the probability measure (*µ*), whereas negative values of *q* < − 1 explore lower densities (Caniego et al. 2001; Chhabra and Jensen 1989).

For 3D objects for which the monofractal assumption holds, the multifractal spectra simplify as follows. The *f*(*α*) singularity spectrum is reduced to a point at maximum value *f*(*α*_*0*_), and *D*_*q*_ is a constant function of *q*, i.e., the Rényi spectrum is represented by a horizontal line.

## Results

### Pore morphology of softwood pellet biochar

**Fig. 1:**
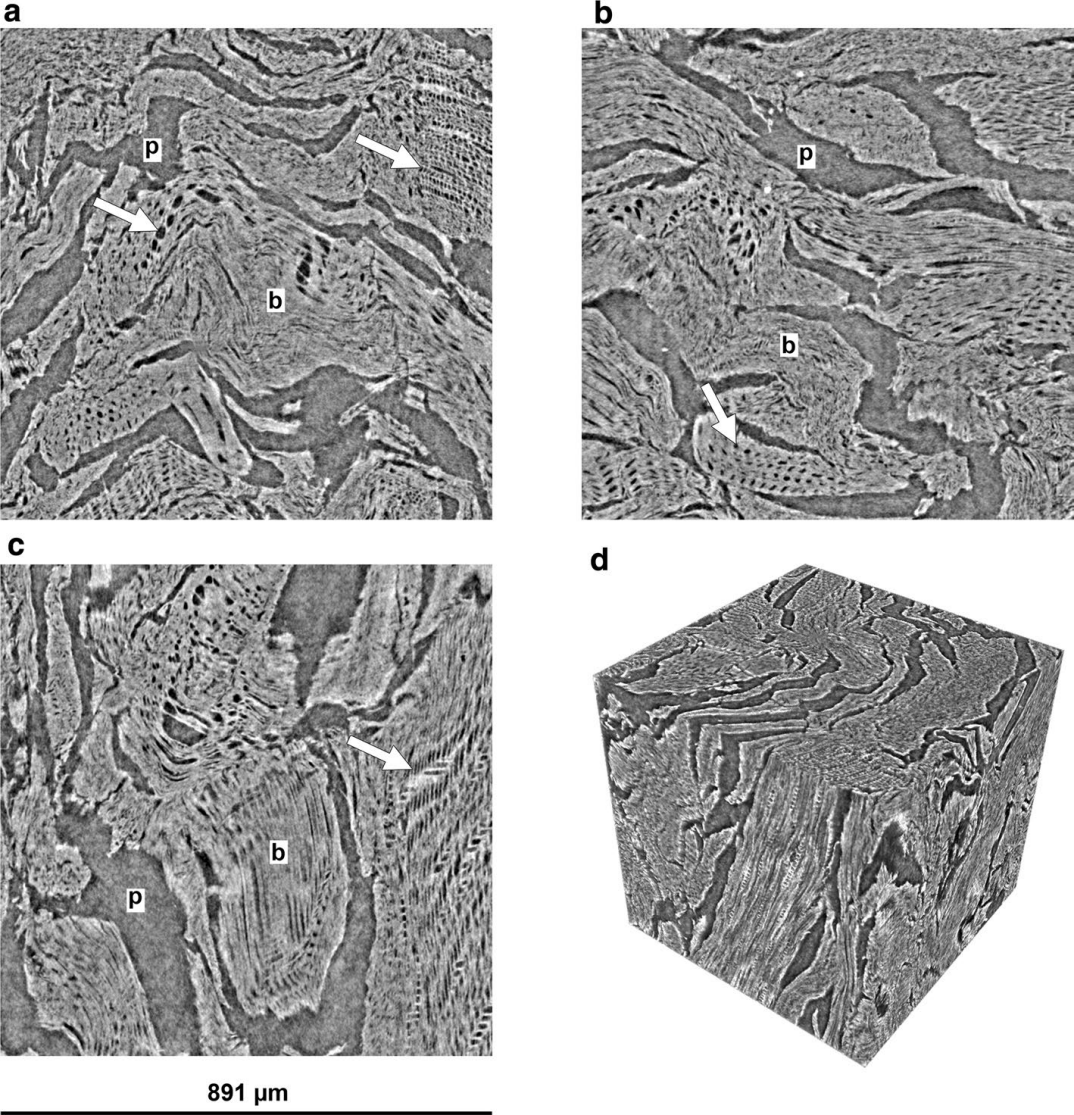
Illustrations of the SWP550 biochar structure, as reconstructed from X-ray microtomography data. Presented are orthogonal cross sections (**a** *X*–*Y*-plane; **b** *Y*–*Z*-plane; **c** *X*–*Z*-plane) and a 3D rendering (**d**) of the cropped image cube. The small letters “p” and “b” indicate pore spaces and biochar structures, respectively. Arrows point to areas that had a stronger resemblance to the cellular structures found in softwood

**Fig. 2:**
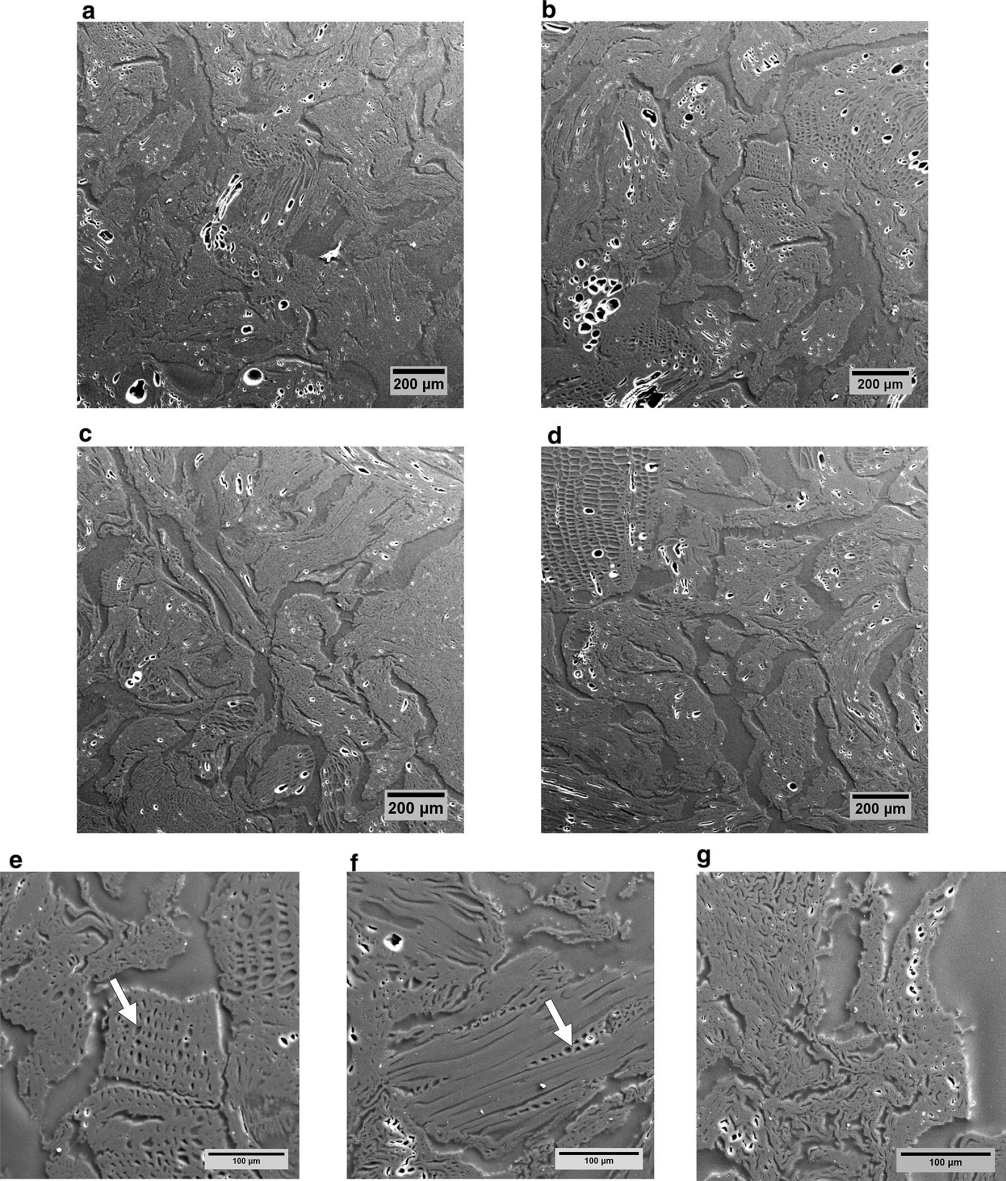
Scanning electron micrographs of resin-embedded softwood pellet biochar (SWP550). Transversal (**a**, **b**) and longitudinal sections (**c**, **d**) of the pellet exhibit very similar structures. Arrows highlight pore structures originating from softwood cells (tracheids (**e**) and rays (**f**)), although overall the wood cellular structure was largely destroyed due to pelleting and thermochemical decomposition during pyrolysis, as shown in insert (**g**)

**Fig. 3:**
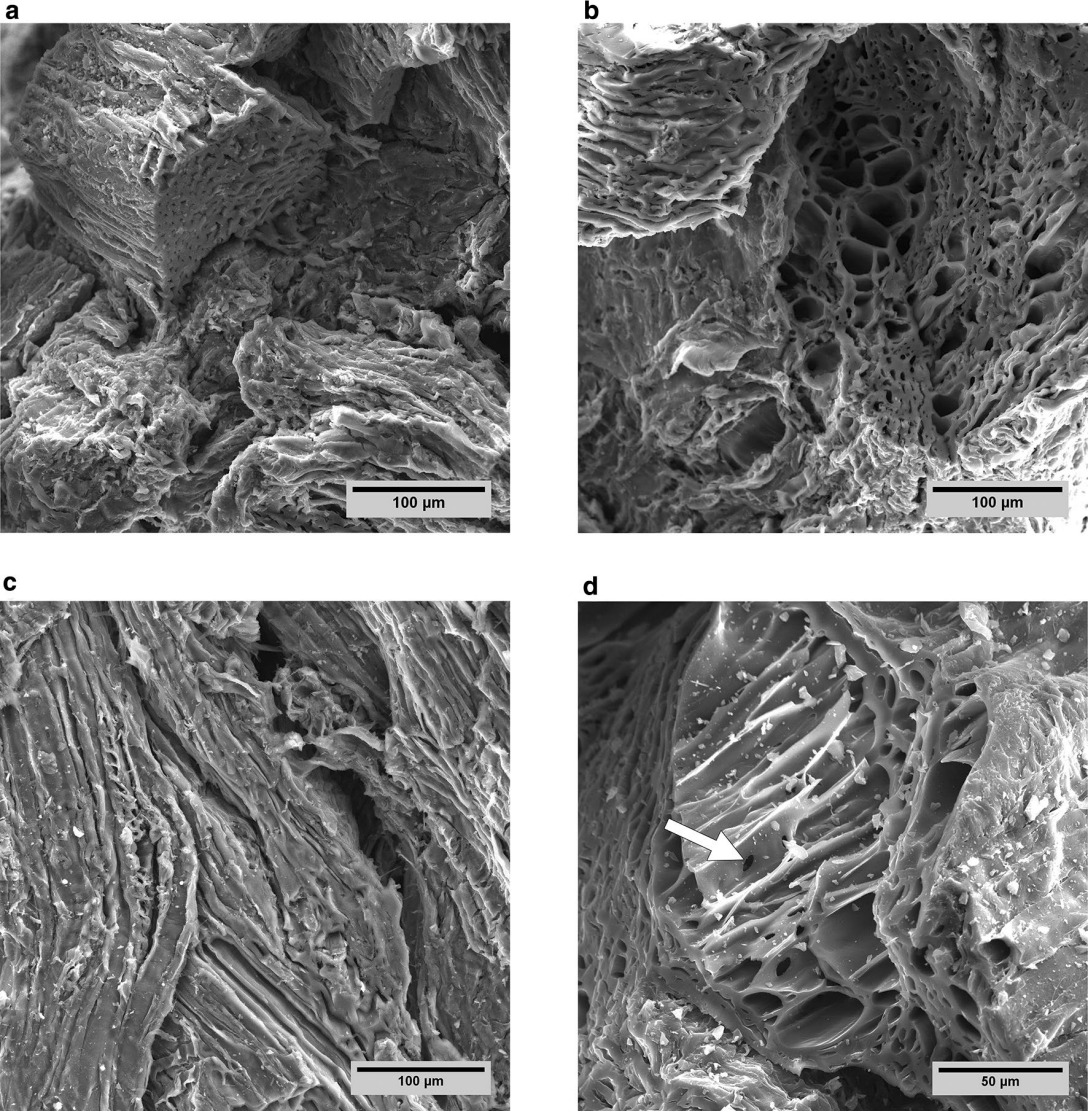
Representative scanning electron micrographs of 1–2 mm fragments of softwood pellet biochar depicting compressed or deformed structures (**a**, **c**), large accessible pores (**b**), and wood cellular structures (**d**) with connecting pores (arrow)

We examined the pore system of softwood pellet biochars by SEM and phase-contrast X-ray microtomography at a voxel resolution of 0.87 µm. The biochar samples exhibited remnants of the original softwood morphology, but pelleting and thermochemical decomposition greatly modified the pore structure. Microtomographic reconstructions for SWP550 replicates, depicted in Fig. 1, showed that the pellets were composed of disarranged wood fragments. As biomass shrunk during pyrolysis, large elongated or slit-shaped pores of approximately 30–150 µm in width formed between separate wood fragments. These large pores pervaded the whole biochar pellet, showed a high level of connectivity, and were also connected to the external surface of the pellet. Two-dimensional orthogonal sections through the reconstructed image (Fig. 1a–c) revealed transverse views of pores derived from wood tracheids. Most of these elongated, tracheid-derived pores were compressed, irregularly shaped, and approximately 10–20 µm wide. Less compressed tracheid pores measured between 25 and 35 µm. For comparison, SEM micrographs of sections through resin-embedded biochar pellets were created and revealed pore structures that are consistent with those from microtomography (Fig. 2). The structures in SEM micrographs did not seem to differ between the cross and longitudinal sections and appeared to be very similar throughout the pellet (Fig. 2a–d). As in the micrographic reconstructions, pores derived from softwood cells are recognizable (Fig. 2e, f); however, most pores were structurally altered and do not resemble any cellular features of softwood (Fig. 2g). In addition, the surface topography of 1–2 mm fragments of pellet biochars were examined by SEM, and the micrographs illustrate a rugged surface with accessible pores and slits (Fig. 3). Again, deformed or flattened tracheid structures are discernible, and these are connected through openings in their walls (Fig. 3d).

### Quantitative analysis of biochar porosity

**Fig. 4:**
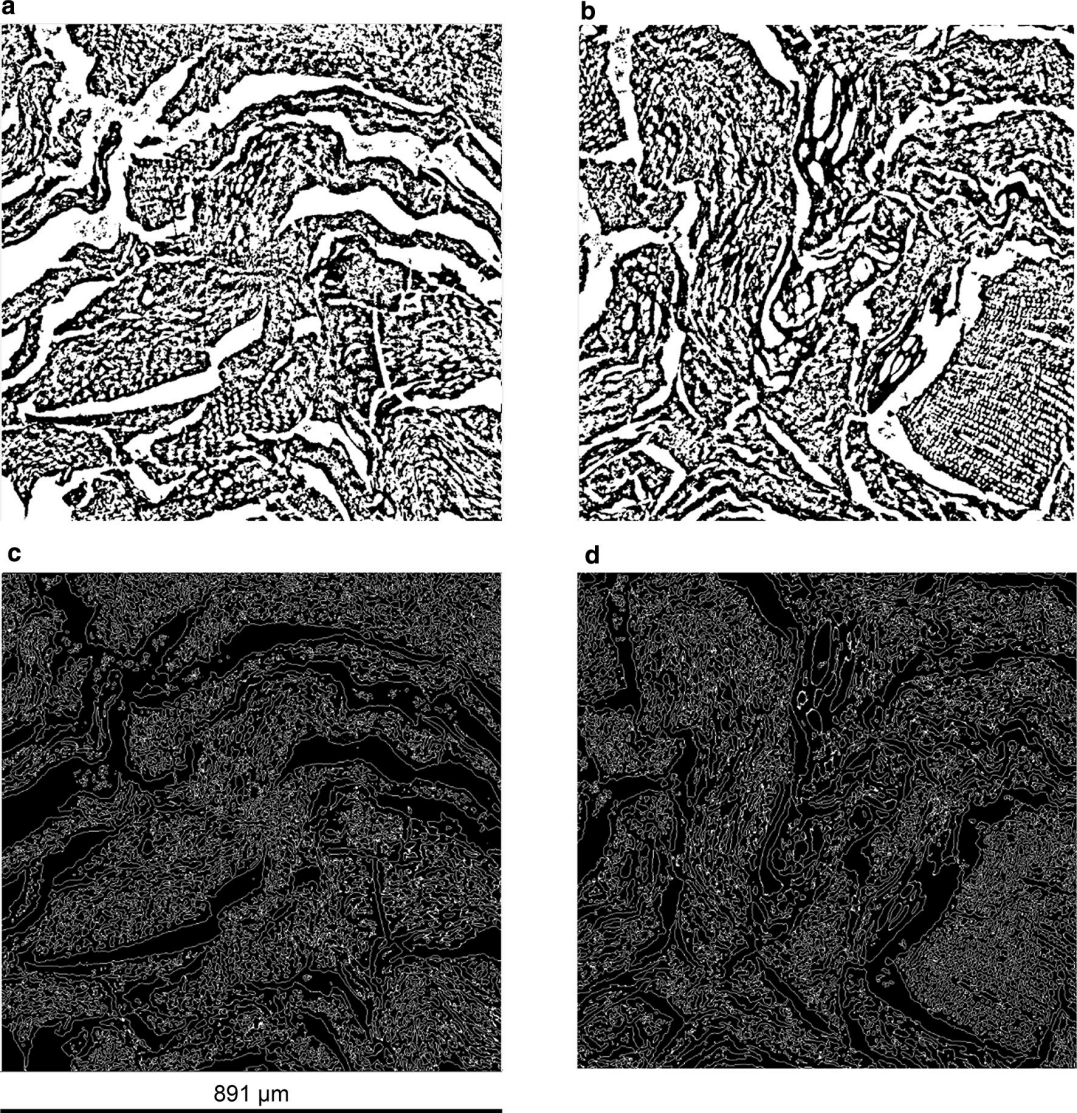
Segmentation of biochar pores from X-ray tomography data. Representative image slices of the region of interest (1024^3^ voxels) for SWP550 (**a**) and SWP700 (**b**) after segmentation; black voxels: biochar solid; white voxels: pores. The surface of the largest connected pore is represented as white contours in **c** and **d**, which show the same regions of interest as in **a** and **b** for SWP550 and SWP700, respectively

Representative binary images of the segmented biochar structures for SWP550 and SWP700 are shown in Fig. 4, with the biochar solid depicted in black voxels and void space in white voxels. Since the average total porosity in the ROI was 0.58 for SWP550 and 0.575 for SWP700, it is evident that the pyrolysis temperature did not affect the observed total porosity (Table 2). The analysis of pore volume distributions revealed that the pore structure in all samples consisted of one large, connected pore network, which accounted for 99.9% of the total porosity, and numerous much smaller pores that all together followed a negative exponential distribution (Fig. S1). About 95% of these small pores were isolated (closed) and had a volume below 43 voxels, corresponding to a pore width of about 3 µm. Therefore, these pores can be considered to be inaccessible for microorganisms. The shape of the connected pore networks shows a high degree of complexity, as indicated by the very large 0.23 value of average surface-to-volume ratio (Table 2). By comparison, the surface area-to-volume ratio of a sphere with an equivalent volume is 0.0057. The surface of pore networks is depicted as contours in panels c and d of Fig. 4, to illustrate the complexity of the structure and its connectivity throughout the ROI.

**Table 2:** Estimates of total porosity in the region of interest (1024^3^ voxels) and of volume and surface area-to-volume ratio for the largest pore for each replicate sample of SWP550 and SWP700 biochars

Sample	Total porosity (−)	Pore volume fraction of the largest pore (−)	Volume of largest pore (mm^3^)	Surface area-to-volume ratio of largest pore (−)	Surface area-to-volume ratio of sphere with equivalent volume (−)
SWP550-1	0.577	0.9993	0.408	0.242	0.00567
SWP550-2	0.582	0.9991	0.411	0.220	0.00566
SWP700-1	0.565	0.9992	0.399	0.231	0.00572
SWP700-2	0.582	0.9994	0.411	0.235	0.00566
SWP700-3	0.578	0.9992	0.408	0.225	0.00567

The surface area-to-volume ratio of a sphere with volume equivalent to that of the largest pore is given for comparison. The surface area-to-volume ratio was calculated by dividing the surface area (in number of voxels) by the volume of the pore (in number of voxels)

**Fig. 5:**
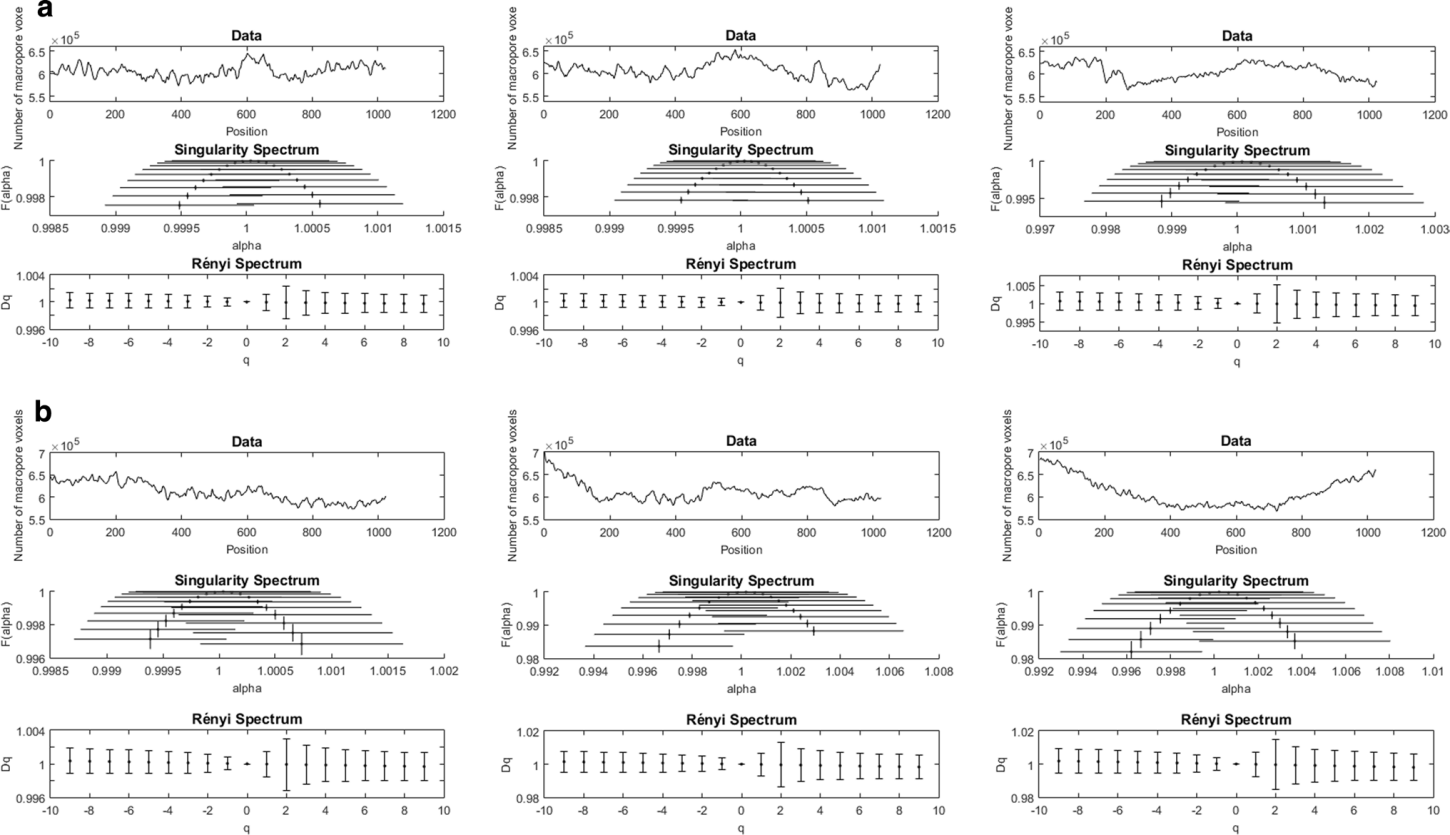
Multifractal analysis results for replicate 1 (**a**) and replicate 2 (**b**) of SWP550 biochar along the three axes of the image cube (left, middle, right). The frequency distribution of pore voxels along one axis of the cube is shown in the top data plot of each panel. The symmetrical shape of the singularity spectrum curves indicates a well-balanced distribution of pore density. The almost horizontal straight line formed by the estimates of generalized dimension *D*_*q*_ in Rényi spectra indicates homogeneity in the complexity of pore network structures. Error bars represent standard errors

**Fig. 6:**
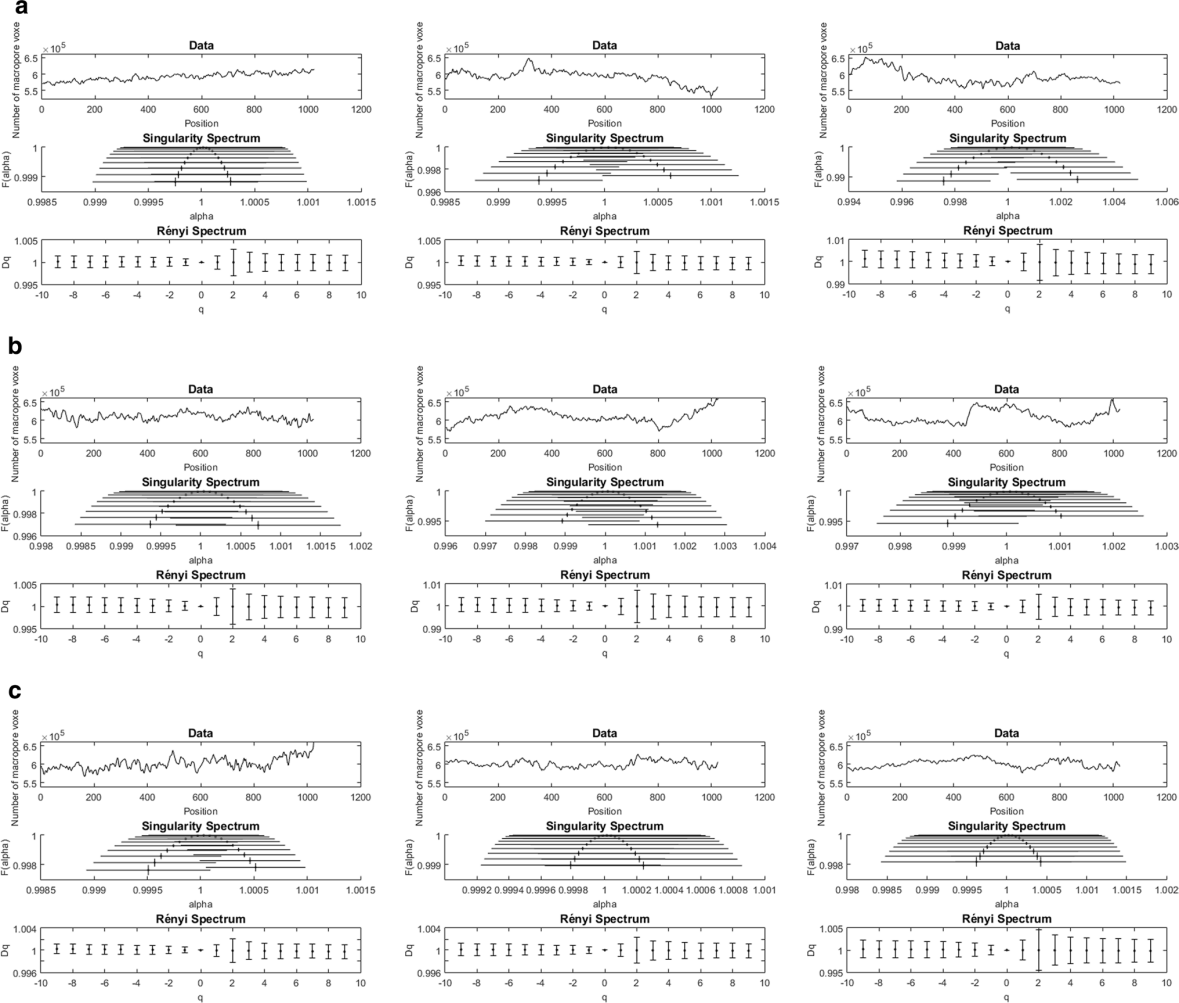
Multifractal analysis results for three replicate particles (**a**–**c**) of SWP700 biochar along the three axes of the image cube (left, middle, right). The frequency distribution of pore voxels along one axis of the cube is shown in the top data plot of each panel. The symmetrical shape of the singularity spectrum curves indicates a well-balanced distribution of pore density. The almost horizontal straight line formed by the estimates of generalized dimension *D*_*q*_ in Rényi spectra indicates homogeneity in the complexity of pore network structures. Error bars represent standard errors

The number of pore voxels in each image slice along the three directions through the ROI cubes of 1024^3^ voxels, was calculated for each of the two replicate samples of SWP550 and the three replicate samples of SWP700. The resulting pore frequency curves, presented in the top panels of Fig. 5 (SWP550) and Fig. 6 (SWP700), fluctuate with relatively small amplitudes around a constant level of approximately 600,000 pore voxels per image slice. The highest porosity value per image slice is 0.67, and the smallest 0.51, across frequency curves. The fact that the pore frequency curves show little variability between orientations for each volume gives a first indication for isotropic porosity distribution. By contrast, cellular structures of wood and wood-derived biochars are anisotropic, and thus, different patterns in porosity frequency curves along the longitudinal, radial and tangential axes would be expected in that case.

### Multifractal analysis of pore distribution in softwood pellet biochar

Based on the pore frequency curves, multifractal analysis was performed with the aim of characterizing the spatial distribution of pore voxels in the segmented 3D images of biochars. The resulting singularity and Rényi spectra are presented in Figs. 5 and 6 for SWP550 and SWP700, respectively. The multifractal parameter estimates obtained from these spectra are given in the Supplementary Material (Tables S1, S2).

In a singularity spectrum, the estimated fractal dimensions *f*(*α*_*q*_) are plotted against the corresponding estimated singularity exponents *α*_*q*_, with the corresponding standard errors. The narrow width and height of all the singularity spectra, which almost collapse to a single point: *α* = 1 and *f*(*α*) = 1, implies that there was very little heterogeneity in the distribution of porosity and almost constancy in its complexity. The symmetrical shape of the concave downward curves [*A* = (*α*_0_ − *α*_min_)/(*α*_max_ − *α*_0_) ≈ 1] indicates that regions with lower densities (negative *q*) and higher densities (positive *q*) of pores scaled similarly. This basically means that the distribution of porosity in SWP biochar exhibits monofractal scaling behavior.

The multifractality of pore distribution for each biochar sample was also assessed using the generalized dimensions *D*_*q*_ in Rényi spectra. For a multifractal, *D*_*q*_ changes with *q* (e.g., *D*_*−*2_ > *D*_*−*1_ > *D*_0_ > *D*_1_ > *D*_2_), whereas *D*_*q*_ of a monofractal remains essentially unchanged for all *q*. In our study, the estimated values for *D*_*q*_ form an almost horizontal straight line. More specifically, the estimates of *D*_1_ and *D*_*2*_ are very close to the capacity dimension *D*_0_ (standardized to 1.0), and the differences (*D*_0_ − *D*_2_) and (*D*_*q* min_ − *D*_*q* max_) are very small. Consistent with the results from the singularity spectra, all of this leads to the conclusion that the pore distribution is very homogeneous and monofractal instead of multifractal. Moreover, the results of multifractal analysis did not reveal any significant differences in the shapes of spectra between the three directions of the image cubes. This indicates that the spatial distribution of pores in the analyzed biochar volumes is isotropic, i.e., invariant with respect to the rotation of the sample.

## Discussion

Synchrotron X-ray microtomography and image analysis provide a powerful approach for the investigation of biochar pore structures. Subsequent multifractal analysis performed on reconstructed, segmented 3D images of pores enables the qualitative characterization of the pore distribution in the 3D biochar matrix. The information describing the complex microstructural geometry is then summarized in multifractal parameters, which can be compared between multiple data sets.

In this study, X-ray phase-contrast microtomography imaging was used to enhance the contrast on edges between carbon-based solid matrix and air-filled pores. This technique has been previously shown to be suitable for uniform materials with low X-ray absorption (Mayo et al. 2010). With an image resolution of 1.74 µm, we were able to account for biochar porosity that plays a role in soil hydrological processes, plant growth and microbial colonization. We studied the distribution of porosity in a sub-volume of the SWP biochar particle through multifractal analysis performed on porosity frequency curves along the 3 dimensions of the image volume. The distribution of pores in biochar exhibited a rather monofractal behavior, which was reflected in the shapes of multifractal spectra. The curves in Rényi spectra had a near linear shape, and the curves in singularity spectra were symmetrical, narrow arcs around *f*(*α*_0_) = 1. This implies that the pore structure in the analyzed volume was homogeneous with little fluctuation of high or low porosity and could be described almost entirely by the capacity dimension *D*_0_ standardized to 1. Based on the high similarity of pore frequency curves and multifractal parameters from the analysis of pore distribution along the three directions of the image cube, we concluded that the porosity distribution was isotropic within the biochar particles. By contrast, the pore structure of raw wood has a high degree of anisotropy, originating from the axially oriented vascular tissue of the tree, which is preserved during production of biochar (Pattanotai et al. 2014).

Depending on the quality of the feedstock, there can be significant variation in the micrometer-scale pore structures of biochars, which may affect their suitability for applications in which the porosity plays an important role. In the case of wood biomass, for example, earlywood and latewood differ in density and pore size, sapwood and bark exhibit very different pore structures, and there are large differences in cell morphology between tree species (Ciesielski et al. 2015; Hyväluoma et al. 2018b). The high natural variability of biomass feedstocks, with regard to their physical properties and chemical composition, has an impact on the performance of thermochemical biomass conversion processes, such as pyrolysis (Williams et al. 2016). To ensure homogeneous physical properties, particle size reduction and biomass densification are required. For the production of pellets, wood (or other biomass) is milled and screened to particle size of typically < 3 mm, before it is densified into cylindrical pellets in a pellet mill, thereby increasing the biomass bulk density about four to five times that of the loose material (Tumuluru et al. 2011; Yancey et al. 2013). Pecha et al. (2018) examined the internal structure and porosity of milled pine wood particles and fragments of a crushed pine wood pellet using X-ray microtomography. The authors determined an intraparticle porosity of 0.65 for non-densified and 0.27 for pelletized pine from microtomographic images at a 3.4 µm voxel resolution. The reconstructed structure of the pelletized particle presented by Pecha et al. (2018) had a remarkable resemblance to the internal morphology of softwood pellet biochar revealed in our study. It was evident that the pelleting process destroyed most of the cellular structure and porosity, creating a tightly and randomly packed mass of smaller particles. Nevertheless, the shrinkage of biomass during slow pyrolysis caused the formation of large, elongated pores between wood fragments, resulting in an observed porosity of 0.577 ± 0.007 across all SWP biochar samples (*n* = 5). Although one has to be cautious when comparing image analysis data from different studies, due to differences in image resolution and procedures for image processing and segmentation, it appears that the observed porosity values fall within a range of porosities reported for other softwood-derived biochars. Porosity values of 0.55 and 0.6 were recorded for non-densified Scots pine biochar produced by slow pyrolysis at 475 °C, based on microtomographic images at 1.14 µm voxel resolution (Hyväluoma et al. 2018b). Reconstructed 3D images of biochars produced from used softwood pallets by slow pyrolysis at 400 and 700 °C had pore volume fractions of 0.5 and 0.51, respectively (Berhanu et al. 2018). Therefore, it seems that densification does not cause a significant reduction in micrometer-scale porosity in pellet biochar, compared to raw wood biochars. From the investigation of binarized 3D images of biochars pore structures, it was found in each sample that 99.9% of the observed porosity was contained in one connected network of pores exhibiting a complex structure with a large surface area. It is conceivable that this network of pores can enable the exchange of water, nutrients and gases, and provide accessible sorption sites as well as habitable spaces for microorganisms.

Pyrolysis conditions can to some extent influence the pore structure in wood-derived biochar. Congruent with earlier reports establishing that higher temperatures promoted the formation of micropores but do not alter the macroporosity (Berhanu et al. 2018; Brewer et al. 2014; Hyväluoma et al. 2018a), the highest treatment temperature did not affect total observed porosity in this study, but a higher temperature resulted in an increase in BET surface area from 26.4 for SWP550 to 162.3 m^2^ g^−1^ for SWP700. High heating rates (> 100 °C min^−1^), as are typical in fast pyrolysis, can significantly influence the pore structure of wood biochar. Pattanotai et al. (2014) demonstrated that after pyrolysis with a heating rate of 60 °C min^−1^ to a maximum temperature of 900 °C and holding time of 5 min, the structure in Japanese cypress wood stayed largely intact with pores mainly in the range of 10–20 µm, whereas a heating rate of 1800 °C min^−1^ resulted in a substantial breakdown of the pore structure, associated with an expansion or collapse of pore walls, due to high internal pressure during pyrolysis. Therefore, slow pyrolysis with lower heating rates (< 100 °C min^−1^) and prolonged residence times in the reactor is preferred for the achievement of high yields of biochar, with higher carbon content, stability and intact surface morphology (Mohanty et al. 2013). The SWP biochars investigated here were produced using a pilot-scale rotary kiln under a highly controlled heating regime, as described in detail by Mašek et al. (2018). The authors established that slow pyrolysis conducted with appropriate control of process parameters (heating rate, HTT, residence time at HTT) yielded biochars with consistent quality across different reactor systems, at scales ranging from tens of grams to tens of kilograms per hour of biochar. Our work demonstrated that SWP biochar is also isotropic and homogeneous with regard to its micrometer-range pore structure at particle scale. Since there was little variation in pore characteristics between replicate particles, there is reason to believe that the structure observed in a particle did not depend on the position within the pellet. Therefore, the analyzed particles were representative for the entire biochar pellet, and the pellets constitute a homogeneous bulk material. Hence, biochar production can be increased to industrial scale without diminishing important biochar properties, if the variability in the biomass feedstock is minimized in terms of physical properties and composition.

An important function of biochar as a soil amendment is to improve soil moisture retention capacity and the provision of plant available water by modifying the soil porosity (Edeh et al. 2020; Omondi et al. 2016). Liu et al. (2017) demonstrated that the internal pores of wood biochar particles > 0.251–2.0 mm have the capacity to increase the amount of water retained at field capacity, permanent wilting point, and plant available water in sandy soil. Their results further suggest that larger particle sizes retain more of the internal porosity and that grinding to finer particle sizes could remove pore sizes that store plant available water (0.2–30 µm). In support of the hypothesis that micrometer-scale pores in biochar can directly influence soil water holding capacity, Rasa et al. (2018) established a link between the pore characteristic of willow biochar, which were determined based on X-ray microtomography at 1.14 µm voxel resolution, with water retention properties of clay soil. The researchers found that the willow biochar, produced at 320 °C, affected the soil water retention mainly at pore sizes around 5–10 and 25 µm equivalent pore diameter, which correspond to biochar pores resulting from wood fibers and vessels, respectively. The 3D reconstructions of SWP biochars illustrate that the pore systems contained elongated pores of 30–150 µm in width, which were inter-connected and also connected to the particle surface. These pores could act as transmission pores for the transport of water through the biochar particles with no preferred direction, thereby enhancing the hydraulic conductivity. During dryer periods, these pores drain easily and allow gas exchange. We also observed smaller pore sizes between 10 and 35 µm in SWP biochars, which can store plant available water. Moisture retained in biochar particles is released into surrounding soil when soil is drying out, as Wang et al. (2019) visualized by neutron tomography for 2 mm softwood biochar particles mixed into sand. Therefore, we can speculate that SWP biochar has the potential to improve soil moisture retention properties beneficial for healthy plant growth. SWP biochar may even be applied as entire pellets, as was done in a field experiment by Andrenelli et al. (2016), which showed that the addition of biochar pellets (5 mm diameter and 16 mm length) significantly increased the plant available water capacity in a silty clay loam soil.

Studies by Yu et al. (2016) and Zhou et al. (2018) using microtomography data at 3.7 µm voxel resolution, revealed that wood chip biochar increased the porosity and permeability of macroaggregates in clayey soils. The researchers also determined, based on water flow simulations in reconstructed 3D pore structures, that biochar increases anisotropy and reduces tortuosity of water flow through soil macroaggregates. This could promote the transport of water and nutrients in a specific direction, mainly facilitated by pores > 50 µm, which form between soil and biochar particles.

Modifications of size and spatial distribution of pores in soil aggregates due to biochar may also result in changes in bioaccessible porosity, i.e., the fraction of porosity that is accessible for microorganisms, which carry out important functions in the soil, for instance the degradation of organic pollutants (Akbari and Ghoshal 2015; Akbari et al. 2016). Consequently, if a larger volume of pores are accessible for certain microbes, a larger amount of organic compounds entrapped in these pores can potentially be degraded. Thus, the analysis of bioaccessible pore space in biochar or biochar-amended soil through X-ray microtomography can provide valuable information that could be linked to biodegradation activity.

Wood-derived biochars with defined and homogeneous macropore characteristics are relevant not only to the use as soil conditioner, but also other applications that require a high degree of specification, e.g., the use as adsorbents and catalysts (Liu et al. 2015; Nanda et al. 2015), or as support material in biofilter systems. Biochar produced from wood chips has been proposed to be a suitable biofiltration medium due to its low ash content and bulk density, high carbon content, good moisture retention capacity, high degree of aromaticity and macropore structure (Baltrėnaitė et al. 2017). Laboratory studies by Das et al. (2019) and La et al. (2018) revealed that efficient removal of H_2_S or methane can be achieved with compost-based biofilters amended with spruce or sawdust biochar, respectively. Biochar being reasonably rigid, low in nutrients and inert to microbial degradation, can improve the structural stability of natural filter beds, prevent compaction and excessive accumulation of biomass, which often results in a reduction of porosity, inhomogeneous gas diffusion, and pressure loss (La et al. 2018; Morgan-Sagastume et al. 2001). In line with this, the uniform and highly connected pore system of SWP biochar could facilitate a homogeneous distribution of water, nutrients and gases to microorganisms immobilized on its surface. The larger specific surface area of SWP700 has also the potential to act as adsorbent for pollutants. The larger particle size and regular shape of biochar pellets could be beneficial to prevent pressure loss (Pugliese et al. 2013). A lot of research has been focused on optimizing biochar properties and engineering of biochar (Wang et al. 2020), and these results show that feedstock densification has the potential to become an important tool for tuning biochar properties.

## Conclusions

We were able to visualize and analyze the micrometer-scale pore structure of softwood pellet biochars with a spatial resolution of 1.74 µm, using synchrotron X-ray phase-contrast microtomography. Three-dimensional reconstructions of biochar particles revealed that the pellet consisted of a disarranged agglomeration of wood fragments and elongated 30–150 µm wide pores forming a highly connected network throughout the particle. Pore structures resembling the original cell morphology, typical for softwood, were discernible but mostly deformed.

Multifractal analysis was applied to assess the heterogeneity of the frequency distribution of porosity along the three dimensions of the reconstructed volumes (1024^3^ voxels, 0.707 mm^3^). The symmetrically shaped and narrow curves in singularity spectra and the quasi horizontal straight lines of generalized dimensions in Rényi spectra indicated that the biochar porosity scaling behavior was, in fact, monofractal instead of multifractal. The distribution of pores was homogeneous within the analyzed volumes, and similar among replicate samples. Moreover, the pore distributions were found to be isotropic (direction-independent) in the analyzed volumes, which is in strong contrast with the highly anisotropic (direction-dependent) pore structure of wood.

The highest treatment temperature during slow pyrolysis (550 vs. 700 °C) did not have an effect on the observed total porosity or multifractal parameter estimates. The micrometer-scale pore system in biochar is mainly determined by the quality of the feedstock but can be significantly modified through mechanical pre-treatment, such as pelletizing. The pelleting process creates a dense material with isotropic structure and uniform particle shape to minimize the variability in the biomass feedstock in terms of physical properties and composition. Biochar production by pyrolysis can be increased to industrial scale without diminishing important biochar properties (Mašek et al. 2018). Wood residues from sawmills and other wood processing facilities can serve as valuable feedstock sources for biochar. We demonstrated that unlike raw wood, wood pellets can be converted into porous carbons with isotropic and homogeneous distribution of pores. As these pores are relevant, for example, for the supply of plant accessible water and habitable space for microorganism, our findings combined with the ability to reproduce biochar with such pore distribution offer substantial advantages in various biochar applications and open new promising avenues.

## Supplementary Information

Below is the link to the electronic supplementary material.

Supplementary file1 (DOCX 232 kb)

## Data Availability

Not applicable.

## Code availability

The program for Multifractal Analysis is available on the website of the Environmetrics Laboratory at McGill University https://environmetricslab.mcgill.ca/Programs.html or can be found in the Supplementary Material of Han et al. (2020).
