# Supplementary material for: Synchrotron X-ray microtomography and multifractal analysis for the characterization of pore structure and distribution in softwood pellet biochar
Source: Biochar. 2021 Jun 23;3(4):671–86. doi: 10.1007/s42773-021-00104-3 (PMC8545715; doi:10.1007/s42773-021-00104-3)
Supplement: Supplementary file 1 — Supplementary file1 (DOCX 232 kb) [file 42773_2021_104_MOESM1_ESM.docx]

Supplementary Material

**Synchrotron X-ray Microtomography and Multifractal Analysis for the Characterization of Pore Structure and Distribution in Softwood Pellet Biochar**

Franziska Srocke^a,b^, Liwen Han^a^, Pierre Dutilleul^a^_,_ Xianghui Xiao^c^, Donald L. Smith^a^

and Ondřej Mašek^b^

^a^ Department of Plant Science, Macdonald Campus, McGill University, 21111 Lakeshore Road, Ste-Anne-de-Bellevue, Quebec H9X 3V9, Canada

^b^ UK Biochar Research Centre, School of Geosciences, University of Edinburgh, King's Buildings, Alexander Crum Brown Road, Edinburgh EH9 3FF, United Kingdom

^c^ National Synchrotron Light Source II, Brookhaven National Laboratory, Upton, NY 11973, USA

**Corresponding author:**

Donald L. Smith ([donald.smith@mcgill.ca](mailto:donald.smith@mcgill.ca))

**Supplementary methods: Multifractal analysis**

More detailed descriptions of multifractal analysis can be found in San José Martínez et al. (2010), Lafond et al. (2012) and Han et al. (2020). Following the method by Chhabra and Jensen (1989), the singularity strength *α_q_* and the fractal dimension *f(α_q_)* were defined as

and

,

where the measure *µ_i_*(*q*,*ε*) was given by

.

The singularity strength can be interpreted as a degree of mass concentration of the measure *µ*; the larger *α*, the smaller the concentration of the measure and vice versa. Singularity spectra are created by plotting *f*(*α*) vs. *α*, and display downward parabolic curves with maximum value *f*(*α_0_*) at *α_0_*. Several parameters can be extracted from these curves to obtain information about the characteristics of pore distribution: the width (Δ*α = α_max_*- *α_min_*), asymmetry (*A* = (*α_0_ - α_min_*)/(*α_max_ - α_0_*)), and the extent of the left and right arm of the curves (Δ*f* = *f*(*α_min_*)*- f*(*α_max_*)). The wider the spectrum, the larger is the heterogeneity in singularity strength *α,* and the higher is the complexity of the measure. A broader left arm of the curve (*A* > 1) indicates that there are larger local fluctuations for higher concentrations of the measure (corresponding to positive *q*).

Additionally, values for the generalized dimension *D_q_* were computed according to Grassberger (1983), for *q* ≠ 1

,

and for *q* = 1

.

The generalized dimension curves or Rényi spectra of multifractals generally have a sigmoidal shape with decreasing *D_q_* values for increasing *q*. The greater the variation of values of *D_q_* in the Rényi spectrum (*D_q min_ – D_q max_*), the higher is the degree of heterogeneity of the measure. The *D_q_* value at *q* = 0 is known as the capacity dimension and provides information about the geometric support of the measure, and is the same as *f*(*α_0_*). The *D_q_* value at *q* = 1 is called the information or entropy dimension and can be interpreted as an index of heterogeneity in the distribution of the measure. A *D_1_* value close to 1 indicates evenness of measures over the sets of cell size. The correlation dimension *D_2_* expresses the scaling behavior and measures the average distribution density of the measure.

References:

Chhabra A, Jensen RV (1989) Direct determination of the f(α) singularity spectrum. Phys. Rev. Lett. 62:1327–1330. https://doi.org/10.1103/PhysRevLett.62.1327

Grassberger P (1983) Generalized dimensions of strange attractors. Phys. Lett. A 97*:*227–230. https://doi.org/10.1016/0375-9601(83)90753-3

Han L, Srocke F, Masek O, Smith DL, Lafond JA, Allaire S, Dutilleul P (2020) A Graphical-User-Interface application for multifractal analysis of soil and plant structures. Computers and Electronics in Agriculture 174:105454. https://doi.org/10.1016/j.compag.2020.105454

Lafond JA, Han L, Allaire SE, Dutilleul P (2012) Multifractal properties of porosity as calculated from computed tomography (CT) images of a sandy soil, in relation to soil gas diffusion and linked soil physical properties. Eur. J. Soil Sci. 63:861–873. https://doi.org/10.1111/j.1365-2389.2012.01496.x

San José Martínez F, Martín MA, Caniego FJ, Tuller M, Guber A, Pachepsky Y, García-Gutiérrez C (2010) Multifractal analysis of discretized X-ray CT images for the characterization of soil macropore structures. Geoderma 156:32–42. https://doi.org/10.1016/j.geoderma.2010.01.004


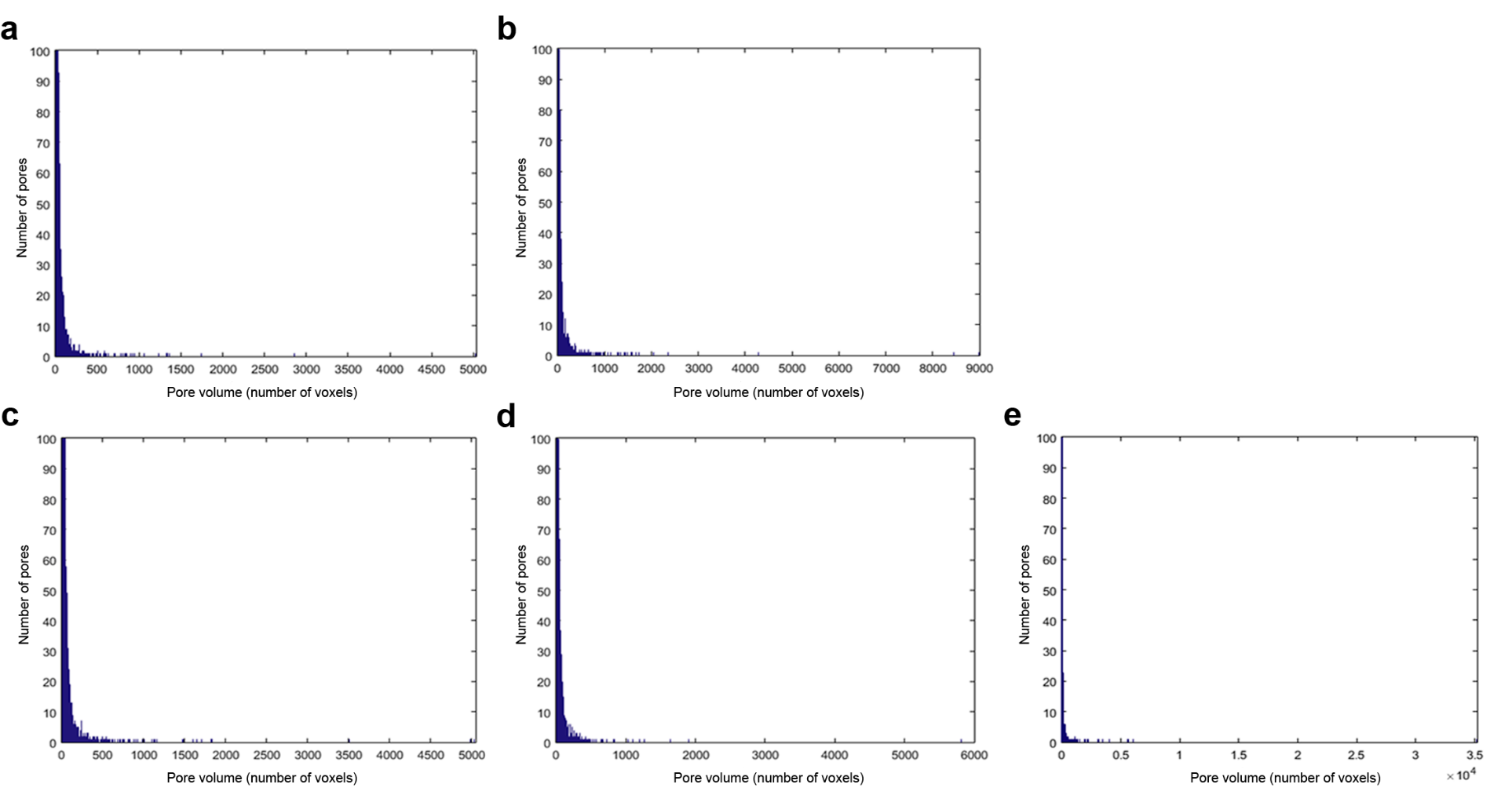


**Fig. S1** Pore volume distributions in regions of interest (1024^3^ voxels) of replicate samples of SWP550 (a,b) and SWP700 (c-e) biochars. For a better presentation of the distributions, the pore with the greatest volume was excluded from each plot, and the y-axes were truncated at a value of 100. The volume of the largest pore was approximately: a) 6.19 × 10^8^ voxels, b) 6.25 × 10^8^ voxels, c) 6.06 × 10^8^ voxels, d) 6.24 × 10^8^ voxels, e) 6.2 × 10^8^ voxels. The maximum values of the number of pores with a volume of 1 voxel were: a) 16207, b) 15405, c) 15278, d) 11887, e) 11525.

| **Table S1** Multifractal parameter estimates from Singularity spectra *f(α)* vs. *α,* for SWP550 and SWP700 biochar samples, for each orientation of the image cube (x, y, z). | | | | | | | | |
| --- | --- | --- | --- | --- | --- | --- | --- | --- |
| **Sample** | **α_max_** | **f(α_max_)** | **α_min_** | **f(α_min_)** | **α_max_ - α_min_** | **α_0_** | **(α_0_ - α_min_)/(α_max_ - α_0_)** | **f(α_min_) - f(α_max_)** |
| **SWP550** |  |  |  |  |  |  |  |  |
| Rep 1-x | 1.00056 | 0.99762 | 0.99949 | 0.99755 | 0.00107 | 1.00003 | 1.02334 | -0.00007 |
| Rep 1-y | 1.00051 | 0.99783 | 0.99955 | 0.99785 | 0.00096 | 1.00003 | 0.99119 | 0.00003 |
| Rep 1-z | 1.00126 | 0.99464 | 0.99891 | 0.99485 | 0.00235 | 1.00007 | 0.97171 | 0.00020 |
|  |  |  |  |  |  |  |  |  |
| Rep 2-x | 1.00074 | 0.99684 | 0.99939 | 0.99713 | 0.00135 | 1.00004 | 0.93029 | 0.00029 |
| Rep 2-y | 1.00293 | 0.98826 | 0.99665 | 0.98366 | 0.00628 | 1.00018 | 1.28167 | -0.00460 |
| Rep 2-z | 1.00368 | 0.98516 | 0.99621 | 0.98194 | 0.00747 | 1.00022 | 1.15983 | -0.00323 |
|  |  |  |  |  |  |  |  |  |
| **SWP700** |  |  |  |  |  |  |  |  |
| Rep 1-x | 1.00027 | 0.99884 | 0.99975 | 0.99882 | 0.00052 | 1.00001 | 1.01107 | -0.00002 |
| Rep 1-y | 1.00062 | 0.99742 | 0.99938 | 0.99700 | 0.00124 | 1.00003 | 1.12156 | -0.00043 |
| Rep 1-z | 1.00263 | 0.98902 | 0.99757 | 0.98848 | 0.00506 | 1.00014 | 1.03685 | -0.00053 |
|  |  |  |  |  |  |  |  |  |
| Rep 2-x | 1.00072 | 0.99693 | 0.99937 | 0.99701 | 0.00135 | 1.00004 | 0.98055 | 0.00008 |
| Rep 2-y | 1.00131 | 0.99431 | 0.99893 | 0.99496 | 0.00239 | 1.00007 | 0.91356 | 0.00065 |
| Rep 2-z | 1.00102 | 0.99584 | 0.99889 | 0.99459 | 0.00213 | 1.00006 | 1.21890 | -0.00125 |
|  |  |  |  |  |  |  |  |  |
| Rep 3-x | 1.00051 | 0.99784 | 0.99951 | 0.99763 | 0.00101 | 1.00003 | 1.07200 | -0.00021 |
| Rep 3-y | 1.00027 | 0.99883 | 0.99976 | 0.99884 | 0.00052 | 1.00001 | 0.99461 | 0.00001 |
| Rep 3-z | 1.00045 | 0.99808 | 0.99959 | 0.99807 | 0.00086 | 1.00002 | 1.00679 | -0.00002 |

| **Table S2** Multifractal parameter estimates from Rényi spectra of SWP550 and SWP700 biochar samples, for each orientation of the image cube (x, y, z). *D_0_*, capacity dimension; *D_1_*, information dimension; *D_2_*, correlation dimension; *D_min_*, *D_q_* at *q* = -9; *D_max_,* *D_q_* at *q* = 9. | | | | | | | |
| --- | --- | --- | --- | --- | --- | --- | --- |
| **Sample** | ***D_0_*** | ***D_1_*** | ***D_2_*** | ***D_0_ - D_2_*** | ***D_min_*** | ***D_max_*** | ***D_min_ – D_max_*** |
| **SWP550** |  |  |  |  |  |  |  |
| Rep 1-x | 1 | 0.99997 | 0.99994 | 0.00006 | 1.00027 | 0.99973 | 0.00054 |
| Rep 1-y | 1 | 0.99997 | 0.99995 | 0.00005 | 1.00024 | 0.99976 | 0.00048 |
| Rep 1-z | 1 | 0.99993 | 0.99987 | 0.00013 | 1.00060 | 0.99942 | 0.00118 |
|  |  |  |  |  |  |  |  |
| Rep 2-x | 1 | 0.99996 | 0.99993 | 0.00007 | 1.00035 | 0.99967 | 0.00067 |
| Rep 2-y | 1 | 0.99982 | 0.99964 | 0.00036 | 1.00146 | 0.99827 | 0.00319 |
| Rep 2-z | 1 | 0.99978 | 0.99956 | 0.00044 | 1.00182 | 0.99799 | 0.00383 |
|  |  |  |  |  |  |  |  |
| **SWP700** |  |  |  |  |  |  |  |
| Rep 1-x | 1 | 0.99999 | 0.99997 | 0.00003 | 1.00013 | 0.99987 | 0.00026 |
| Rep 1-y | 1 | 0.99997 | 0.99993 | 0.00007 | 1.00030 | 0.99968 | 0.00062 |
| Rep 1-z | 1 | 0.99986 | 0.99971 | 0.00029 | 1.00127 | 0.99871 | 0.00256 |
|  |  |  |  |  |  |  |  |
| Rep 2-x | 1 | 0.99996 | 0.99993 | 0.00007 | 1.00034 | 0.99966 | 0.00068 |
| Rep 2-y | 1 | 0.99993 | 0.99987 | 0.00013 | 1.00061 | 0.99942 | 0.00119 |
| Rep 2-z | 1 | 0.99994 | 0.99988 | 0.00012 | 1.00050 | 0.99943 | 0.00107 |
|  |  |  |  |  |  |  |  |
| Rep 3-x | 1 | 0.99997 | 0.99994 | 0.00006 | 1.00025 | 0.99974 | 0.00050 |
| Rep 3-y | 1 | 0.99999 | 0.99997 | 0.00003 | 1.00013 | 0.99987 | 0.00026 |
| Rep 3-z | 1 | 0.99998 | 0.99995 | 0.00005 | 1.00021 | 0.99978 | 0.00043 |
